# The network structure of schizotypy in the general population

**DOI:** 10.1007/s00406-019-01078-x

**Published:** 2019-10-23

**Authors:** Bertalan Polner, Eliana Faiola, Maria F. Urquijo, Inga Meyhöfer, Maria Steffens, Levente Rónai, Nikolaos Koutsouleris, Ulrich Ettinger

**Affiliations:** 1 Department of Cognitive Science, Budapest University of Technology and Economics, Egry József utca 1., T épület, V. emelet 506, Budapest, 1111 Hungary; 2 Department of Psychology, University of Bonn, Kaiser-Karl-Ring 9, 53111 Bonn, Germany; 3 Department of Psychiatry and Psychotherapy, University of Munich, Nussbaumstr. 7, 80336 Munich, Germany; 4 Department of Psychiatry and Psychotherapy, Münster University Hospital, Westfälische Wilhelms-University, Albert-Schweitzer-Campus 1, 48149 Münster, Germany; 5 Institute of Psychology, University of Szeged, Egyetem u. 2, Szeged, 6722 Hungary

**Keywords:** Schizotypy, Schizophrenia, Personality, Network analysis, Factor structure

## Abstract

Schizotypal personality traits show similarity with schizophrenia at various levels of analysis. It is generally agreed that schizotypal personality is multidimensional; however, it is still debated whether impulsive nonconformity should be incorporated into theories and measurement of schizotypy. In addition, relatively little is known about the network structure of the four-dimensional model of schizotypal personality. To estimate the network structure of schizotypy, we used data from participants recruited from the community (*N* = 11,807) who completed the short version of the Oxford-Liverpool Inventory of Feelings and Experiences, a widespread self-report instrument that assesses the positive, negative, disorganised and impulsive domains of schizotypy. We performed community detection, then examined differences between communities in terms of centralities and compared the strength of edges within and between communities. We found communities that almost perfectly corresponded to the a priori-defined subscales (93% overlap, normalised mutual information = 0.74). Items in the disorganisation community had higher closeness centrality relative to items in the other communities (Cliff’s Δs ranged from 0.55 to 0.83) and weights of edges within the disorganisation community were stronger as compared to the negative schizotypy and impulsive nonconformity communities (Cliff’s Δs = 0.33). Our findings imply that the inclusion of impulsive nonconformity items does not dilute the classical three-factor structure of positive, negative and disorganised schizotypy. The high closeness centrality of disorganisation concurs with theories positing that cognitive slippage and associative loosening are core features of the schizophrenic phenotype.

**Electronic supplementary material:**

The online version of this article (10.1007/s00406-019-01078-x) contains supplementary material, which is available to authorized users.

## Introduction

Schizotypal personality traits phenomenologically resemble, at subclinical level, the signs and symptoms of schizophrenia, and they are associated with schizotypy, which has been conceptualised as a latent liability for schizophrenia [1–3]. Schizotypal traits parallel schizophrenia in terms of aetiological risk factors [4–6], profile of cognitive impairments [7–9], and general psychopathology, substance abuse, and suicide attempts [10–12]; additionally, high schizotypy can predict future onset of psychotic disorders [10, 13, 14].

Factor analytic evidence supports the multidimensionality of schizotypal personality. In psychiatric and healthy samples, factor modelling of data from the widely used Schizotypal Personality Questionnaire (SPQ) [15] has revealed positive, negative, and disorganised dimensions [16, 17], which has been corroborated by a recent large-scale cross-national confirmatory study [18]. However, other findings have suggested a four-dimensional model that additionally includes paranoia [19, 20], and that model has also been confirmed in a large cross-national sample [18]. While some questionnaires measure only positive and negative schizotypy [e.g. the Wisconsin Schizotypy Scales (WSS) 12, see [21] for a review of instruments], yet others have argued to extend the concept of schizotypal personality with an impulsive nonconformity dimension, similar to Eysenck’s psychoticism concept [22].

The Oxford-Liverpool Inventory of Feelings and Experiences (O-LIFE) is a widespread instrument reflecting a four-dimensional model of schizotypal personality, which includes positive and negative schizotypy, cognitive disorganisation, and impulsive nonconformity [23–25]. However, a study of help-seekers has indicated that impulsive nonconformity might be unstable and likely connected to temporary affective and psychotic symptoms [26]. Accordingly, in large samples from four European countries, confirmatory factor analysis revealed that a three-factor model (positive schizotypy, negative schizotypy, cognitive disorganisation) had the best fit to O-LIFE data [27], in line with the consensus that schizotypy has three components [28]. On the other hand, a few studies have found that three- and four-dimensional models performed comparably well [24, 29]; while in a sample of adolescents, the impulsive nonconformity subscale had only acceptable internal consistency (Guttman’s lambda-2 = 0.59) and 30-day test–retest reliability (ICC = 0.69) [24]. Relatedly, scores on an alternative measure of impulsive nonconformity have been found to predict concurrent psychotic-like and schizotypal experiences and affective symptoms [30], while they did not predict psychosis at a 10-year follow-up [13], suggesting limited predictive validity. Thus, further work is clearly needed on the structure of the O-LIFE, a widely used questionnaire in the schizotypy literature.

A recent addition to the methodological repertoire of psychopathology and personality research is provided by network theory [31, 32]. Network theory emphasises that dynamic interactions between symptoms of mental disorders play a key role in their emergence and maintenance [33], making it straightforward to model mental disorders as complex networks, where nodes represent certain behaviours, cognitions, and emotions, while links represent their interactions [32, 34–36]. Although network theory has frequently been contrasted with the paradigm of assuming common causes behind symptoms of mental disorders and applying latent variable models [32, 34], it has recently been argued that the boundaries between network and common cause models might not be all that clear (see [37] for a detailed discussion): certain network models are mathematically equivalent with certain latent variable models [38], and communities in a network can indicate the presence of latent variables [39]. However, the interpretations of and predictions generated by latent variable and network models differ [40]; for instance, contrary to latent variable models (with local independence assumed) which would predict that intervening on one symptom would not affect another, using a network model one may predict that the effects of intervening on one symptom would spread through the network, causing changes in other symptoms as well [37, 38].

It should be noted that concerns have been raised about the replicability and stability of network models ([41, 42], but also see [43] for an objection); thus, researchers should routinely estimate the stability of network models [44], conduct replication studies in independent samples (e.g. [45]) and follow open science practices [43]. In addition, novel network modelling techniques are likely to provide remedy for some of the concerns [31].

Interactions among multiple symptoms and environmental factors may characterise the formation and maintenance of psychosis [46–49], providing a theoretical foundation for network modelling of the psychosis phenotype. Network studies have shown, for example, that childhood traumas indirectly connect to psychosis symptoms through general psychopathology [50], and that environmental risk factors correlate with stronger connectivity in a transdiagnostic network including psychosis [51]. In adolescents, higher interconnectivity between positive psychotic experiences was associated with previous auditory verbal hallucinations [52]. In a large general population sample, the networks representing the occurrence of positive, negative and disorganised psychotic experiences and their associated impairments were structurally similar, although the impairment network was characterised by significantly stronger connectivity [53].

A network analysis of schizotypy in the general population was recently performed using a large SPQ dataset that was collected in twelve countries [54]. The authors analysed domain-level and item-level networks. Strong connections were observed between domains that were related to the same broader aspect of schizotypy (e.g. positive or negative), and strong connections were also found between items that belonged to the same SPQ subscale. Networks were largely similar across gender and culture (North America vs. China). The rationale of the present study was to provide further information about the relationship between behaviours and experiences that constitute schizotypy, thereby better characterising the structure of the extended psychosis phenotype. We analysed data from a large general population sample using the short version of the Oxford-Liverpool Inventory of Feelings and Experiences (sO-LIFE; [25, 28, 55]), a self-report questionnaire rooted in the personality tradition of schizotypy research [56]. Therefore, the sO-LIFE differs from the SPQ which was created on the basis of the diagnostic criteria of schizotypal personality disorder, and therefore contains more clinically worded items [15, 54]. Moreover, in contrast to a previous schizotypy network study [54], where only a fraction of the sample was from the general population (4251 of 27,001), our entire sample was recruited from the community, and the average age of our sample is higher (30.4 vs. 22.1 years).

Several more recent studies used network modelling to study the structure of schizotypal personality. A study applied exploratory graph analysis to data collected with the Multidimensional Schizotypy Scale (MSS) and its brief version and identified four (disorganised and positive schizotypy plus affective and social anhedonia) and three dimensions (negative, positive and disorganised) on the full and the brief version, respectively [57]. Another study used SPQ data from a general population sample and detected three dimensions: interpersonal, disorganised and cognitive/perceptual, with the latter being the least central in the network [58]. Another study performed the network analyses of the WSS and found that more central items were better predictors of global functioning and schizophrenia spectrum symptoms which were assessed with an interview [59]. Importantly for the present inquiry, the above studies have applied scales that are based on different concepts of schizotypy—none of them reflects the four-dimensional model that incorporates impulsive nonconformity and cognitive disorganisation beyond positive and negative schizotypy.

Therefore, in our study, we characterised the domains of schizotypy with a data-driven community detection algorithm, thereby attempting to conceptually replicate previous factor analytic studies [24, 29]. Additionally, we inferred the core features of schizotypy by examining centralities of items in the network. Given the mathematical equivalence of latent variable and network models [40] and that network communities can indicate latent variables [39], we hypothesised four communities to emerge in the network structure of the sO-LIFE, which parallel the positive, negative, disorganised and impulsive dimensions of the questionnaire. Finally, we conducted exploratory analyses of the obtained community structure: to characterise the position of communities in the network, we compared strength, closeness and betweenness centrality of nodes in different communities; and to assess the coherence of and associations between communities, we compared the strength of edges within and between communities.

## Materials and methods

### Sample

Participants were invited to fill in the questionnaire through an online platform. The online questionnaire was in German and was advertised widely amongst numerous mailing lists and online forums across Germany. No exclusion criteria were applied, but we only retained data from participants who were at least 18 years old. In total, 11,807 participants [3174 (27%) males; mean (age) = 30.4, SD (age) = 10.8, min (age) = 18, max (age) = 81, skewness (age) = 1.13, kurtosis (age) = 0.64] completed the questionnaire.

### Questionnaire

We measured schizotypal traits with the German version [60] of the short Oxford-Liverpool Inventory of Feelings and Experiences (sO-LIFE; [25, 28, 55]). Most items of the O-LIFE are framed to assess normal personality variation related to schizotypy instead of clinically significant manifestations (such as symptoms of schizotypal or schizoid personality disorder), which makes the O-LIFE suitable to examine schizotypy in the general population [60]. The sO-LIFE contains 43 dichotomous items that belong to four subscales: Unusual Experiences (UE; odd perceptual experiences and bizarre beliefs; 12 items), Cognitive Disorganisation (CD; loose associations, difficulties concentrating and social anxiety; 11 items), Impulsive Nonconformity (IN; antisocial and impulsive tendencies; 10 items) and Introvertive Anhedonia (IA; reduced value and enjoyment of physical and social sources of pleasure; 10 items). On each subscale, higher scores indicate higher expression of schizotypy. Test–retest reliability of the subscales were shown to be high (1 month test–retest ≥ 0.69) [21]. In previous samples and in the present study, internal consistency of the short UE and CD subscales was good (*α* ~ 0.8), while the IA and IN subscales had poorer internal consistency (*α* ~ 0.6) [55, 60]. Convergent validity of the subscales has been supported by correlations (*r*’s > 0.26) with subscales of the SPQ that assess the same dimension of schizotypy [24].

### Statistical analysis

Analyses were performed in R [61] [v3.5.0] using RStudio [62] [v1.1.423]. All code and data to reproduce the analyses are provided here: osf.io/epfvq [63]. To estimate the network from the binary sO-LIFE data, we applied the eLasso methodology (for details see [64]) implemented in the IsingFit package [65] [v0.3.1]. The analysis rests on the Ising model: it models pairwise interactions between variables that have two potential states. Practically, the interactions are estimated with multiple logistic regressions where the score (0/1) on each item is predicted from scores on all the other items, and the regression coefficients are regularised with an EBIC optimised lasso method. The hyperparameter *γ* controls the degree of penalty on solutions including more edges. Split-half analyses suggested that *γ* = 1 results in the most stable network. The networks estimated in split-half samples with *γ* = 1 matched the network estimated in the whole sample well: we found strong correlations between their adjacency matrices (median Spearman *ρ* = 0.90, range 0.85–0.94). Stability of network metrics in the whole sample was further investigated with bootstrapping (see Supplementary Material for details).

We visualised the network and calculated node expected influence [66], closeness and betweenness centralities with the qgraph package [67] [v1.4.4], and node predictabilities were computed with the mgm package [68] [v1.2.5]. We detected communities with the fast greedy algorithm [69] implemented in the igraph package [70] [v1.1.2]. Communities are sets of nodes which are more densely connected to each other, as compared to nodes in different communities [69]. The fast greedy algorithm detects communities by directly optimising modularity, a measure which reflects the quality of the division of the network into communities. We chose the fast greedy algorithm for several reasons: it has no tuning parameter as it directly optimises modularity, its deterministic nature facilitates reproducibility, and it tends to return large communities [71]. The latter feature can be considered advantageous for the present application as the sO-LIFE network is relatively small compared to networks analysed in other areas of network science [69, 71]. Nevertheless, this property of the fast greedy algorithm should be kept in mind while interpreting the community structure we report, and we encourage interested readers to try other community detection algorithms on our data. We compared centrality score distributions of communities with the Kruskal–Wallis test, and if it was significant, we applied Mann–Whitney post hoc tests (we calculated the Cliff Δ effect size with effsize package [72] [v0.7.1]. In addition, we compared the absolute weights of edges that connect nodes within communities and also between communities in the same way as it is described above for centralities. These latter metrics differ from node strength, which sums the weight of edges a node has, regardless whether an edge runs within or between communities. Instead, these metrics provide an overall indicator of the strength of edges among nodes that are located within the same community (i.e. the coherence within a domain), and between nodes that are located in different communities (i.e. the connection between domains).

## Results

To facilitate comparison of the sample with other studies, in Table 1, we present the descriptive statistics and correlations characterising sO-LIFE subscale scores.

**Table 1:** Descriptive statistics of and correlations between sO-LIFE subscale scores

	Mean	SD	Median	IQR	Skewness	Kurtosis	*α*	CD	IA	IN
Unusual experiences (UE)	4.12	2.86	4	4	0.41	− 0.72	0.77	0.41/0.41	0.17/0.17	0.39/0.38
Cognitive disorganisation (CD)	5.48	2.96	5	5	0.03	− 0.96	0.77		0.36/0.35	0.48/0.47
Introvertive anhedonia (IA)	2.54	2.04	2	3	0.83	0.17	0.61			0.23/0.22
Impulsive nonconformity (IN)	4.24	2.28	4	3	0.34	− 0.52	0.58			

Descriptive statistics of the sO-LIFE subscale scores in this sample (*N* = 11,807). The last three columns show Pearson *r*/Spearman *ρ* rank correlation coefficients between subscale scores

*SD* standard deviation, *IQR* interquartile range, *α* Cronbach’s alpha

**Fig. 1:**
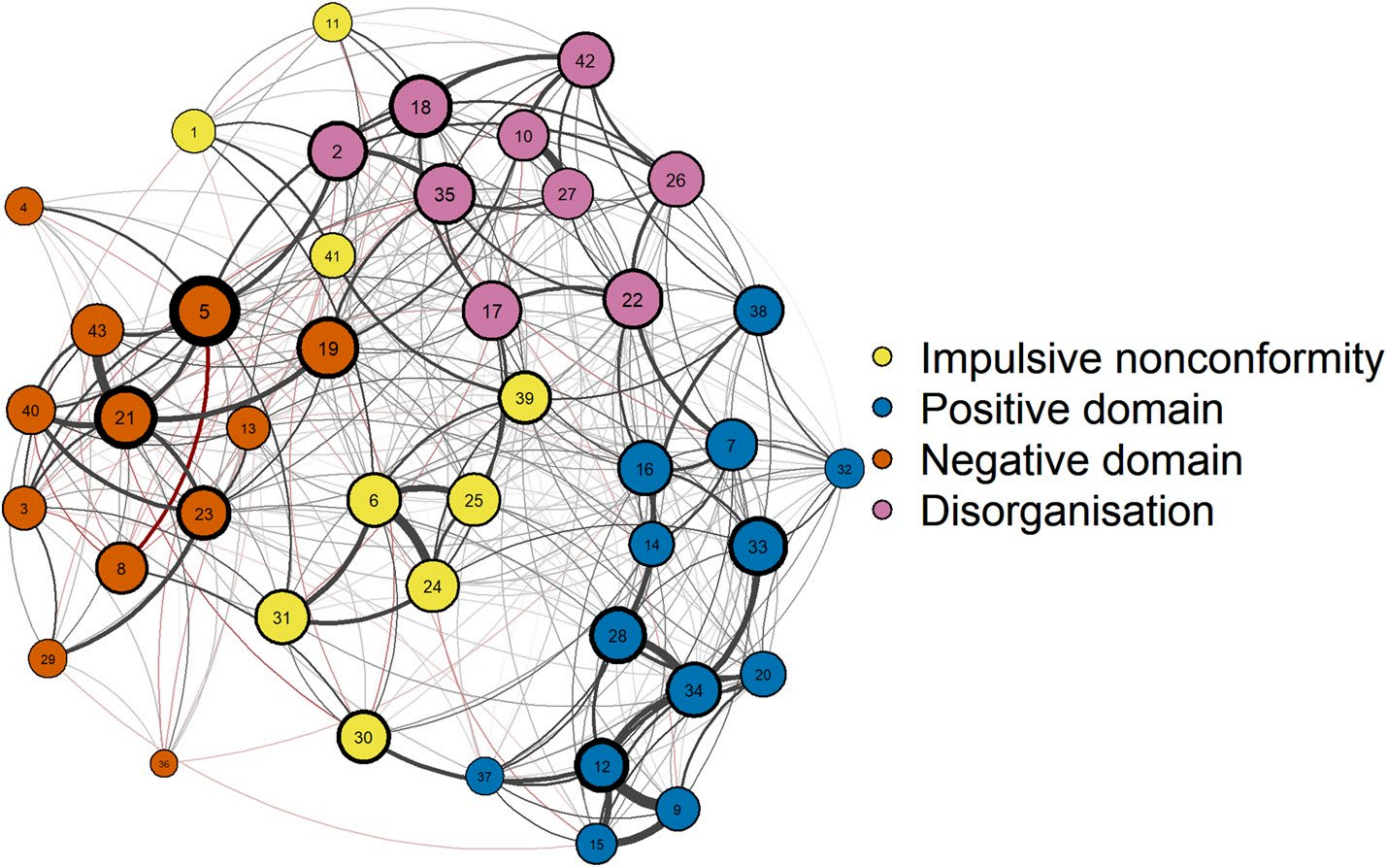
The network structure of schizotypy, as measured with the sO-LIFE in a large online community sample. Nodes represent items and edges represent conditionally independent relationships between items. Node colours indicate communities. Positive edges are shown in grey and negative edges are shown in red. Edge width reflects edge weight, node border width reflects node betweenness, and node size reflects node closeness

The estimated network is shown in Fig. 1. Bootstrapping and split-half analyses both suggested that the estimated network was highly stable in terms of edge weights and node centralities (see Supplementary Material). The fast greedy algorithm detected four communities that almost perfectly overlapped (93% overlap, normalised mutual information = 0.74 [73]) with the a priori-defined subscales of the sO-LIFE (Table 2): impulsive nonconformity (impulsivity, antisocial tendencies and unstable mood), positive domain (hallucination- and delusion-like experiences), negative domain (physical and social anhedonia, and interpersonal difficulties), and disorganisation (poor attention and difficulties in decision making). Community membership did not overlap with the original subscale for only three items (#8, #19, #38).

**Table 2:** Items of the sO-LIFE

#	Item short	Sub	C	#	Item short	Sub	C
1	Alcohol food	IN	Imp	23	Friends touch	IA	Neg
2	Difficulty starting	CD	Dis	24	Urge break smash	IN	Imp
3	Dancing dull	IA	Neg	25	Urge injure yourself	IN	Imp
4	New foods	IA	Neg	26	Distracted daydreams	CD	Dis
5	Enjoy few	IA	Neg	27	Distracted too much happens	CD	Dis
6	Urge harmful shocking	IN	Imp	28	Vague danger	UE	Pos
7	Almost hears thoughts	UE	Pos	29	Massage	IA	Neg
8	Average mood	IN	Neg	30	Average person	IN	Imp
9	Mindreading	UE	Pos	31	Other afraid of you	IN	Imp
10	Difficulty conversation	CD	Dis	32	Mirror face unusual	UE	Pos
11	Thinking before doing	IN	Imp	33	Shapes in the dark	UE	Pos
12	Magical powers	UE	Pos	34	Evil presence	UE	Pos
13	Too independent	IA	Neg	35	Hard to make decisions	CD	Dis
14	Ideas fast	UE	Pos	36	City lights	IA	Neg
15	Aware by thinking	UE	Pos	37	Strong smell	UE	Pos
16	Thought so real	UE	Pos	38	Words mixed up	CD	Pos
17	Mood up and down	CD	Dis	39	Do the opposite	IN	Imp
18	Difficulty keep interested	CD	Dis	40	Close to friends	IA	Neg
19	Dread going into a room	CD	Neg	41	Spend money	IN	Imp
20	Accidents mysterious	UE	Pos	42	Distracted read or talk	CD	Dis
21	Mixing with people	IA	Neg	43	Watch TV or go out	IA	Neg
22	Difficulty controlling thoughts	CD	Dis				

Order of the items of the sO-LIFE in the present study, the subscale they belong to on the sO-LIFE (Sub), and the community they were assigned to by the algorithm in the present study (C)

*UE* unusual experiences, *CD* cognitive disorganisation, *IN* impulsive nonconformity, *IA* introvertive anhedonia, *Imp* impulsive nonconformity, *Pos* positive domain, *Neg* negative domain, *Dis* disorganisation

**Fig. 2:**
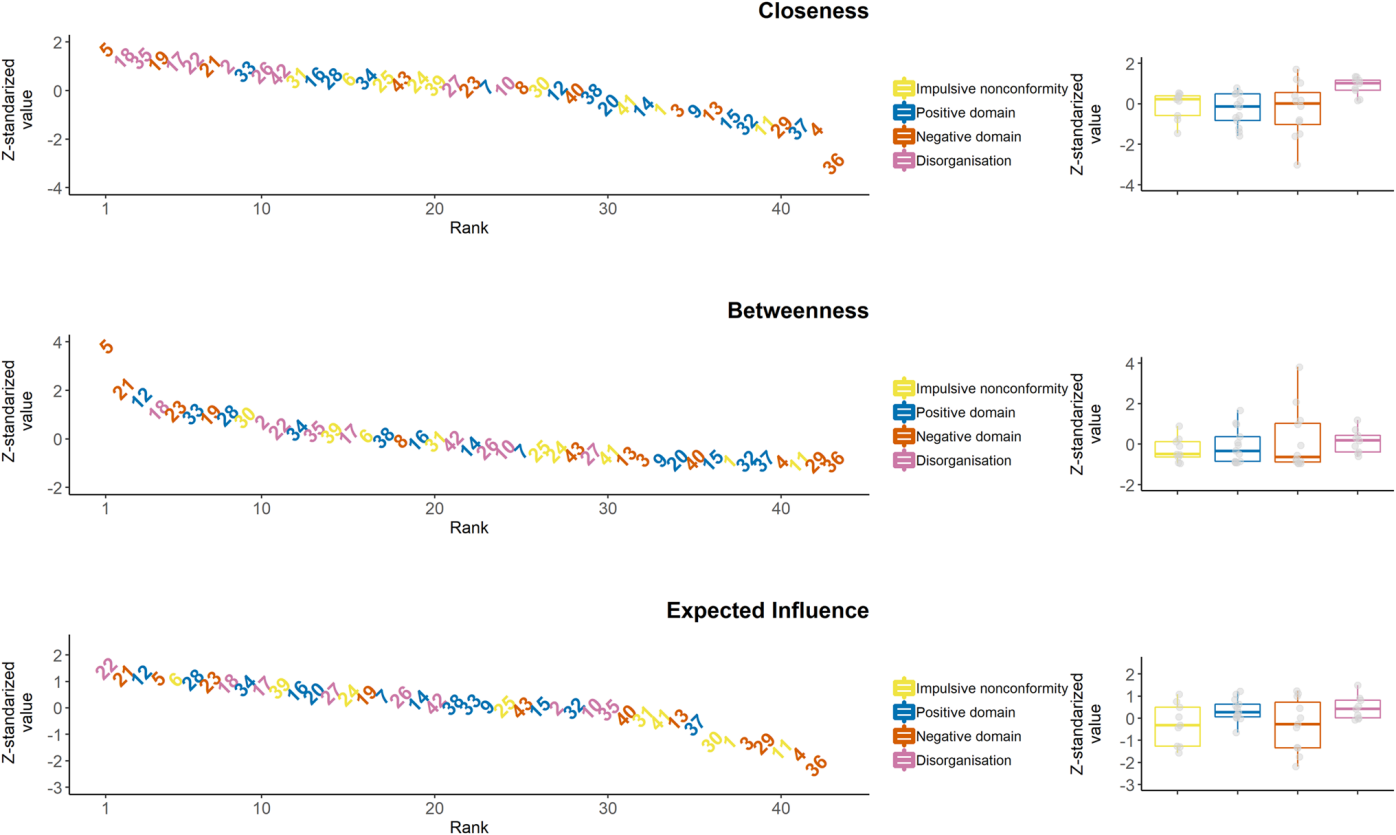
Distribution of *z*-standardised of node closeness, betweenness and expected influence centralities. On the left side of the panels, the nodes are sorted in a descending rank order; while on the right side, the distribution of the *z*-standardised centrality scores are shown by community

Centrality estimates are shown in Fig. 2. Item 5 (enjoy few things) was among the most central according to each of the indices. Bootstrapping difference tests indicated that item 5 had significantly higher centrality scores, relative to almost every other item (see Supplementary Material). Although stability analyses indicated that centralities are reliable, it should be noted that node centralities are prone to sampling variation [44] and therefore, the rank order of item centralities should be interpreted cautiously. Moreover, in the network model of the sO-LIFE, several nodes represent items that assess the same or very similar phenomena with slightly different wording (e.g. #9 mindreading and #12 magical powers), and edges between such nodes might represent the influence of a latent construct [31], making drawing conclusions at the level of items problematic; therefore, we analysed centralities at the community level, and interpret our findings with regard to domains of schizotypal personality represented by the communities. On the other hand, it should be noted that the network included several edges connecting nodes representing items with less overlapping content that were assigned to different communities (e.g. #2 difficulty starting things and #5 enjoy a few things, or #7 almost hearing own thoughts and #22 difficulty controlling thoughts); we suggest that these edges might represent mutualistic interactions.

We compared the centralities of the communities (right side of Fig. 2). Closeness values significantly differed across communities (Kruskal–Wallis *χ*^2^(3) = 10.68; *p* = 0.01). Post hoc tests revealed that closeness of nodes in the disorganisation community differed significantly from closeness of nodes in all the other communities (all Mann–Whitney *p* values < 0.03, Cliff’s Δs ranged from 0.55 to 0.83). Analyses of predictability revealed a similar pattern: nodes in the disorganisation community were significantly more predictable than nodes in the other communities; in addition, nodes in the positive domain community were more predictable than nodes in the negative domain community (see Supplementary Material for details). No significant differences were found for betweenness and expected influence (Kruskal–Wallis *p* values > 0.16).

**Fig. 3:**
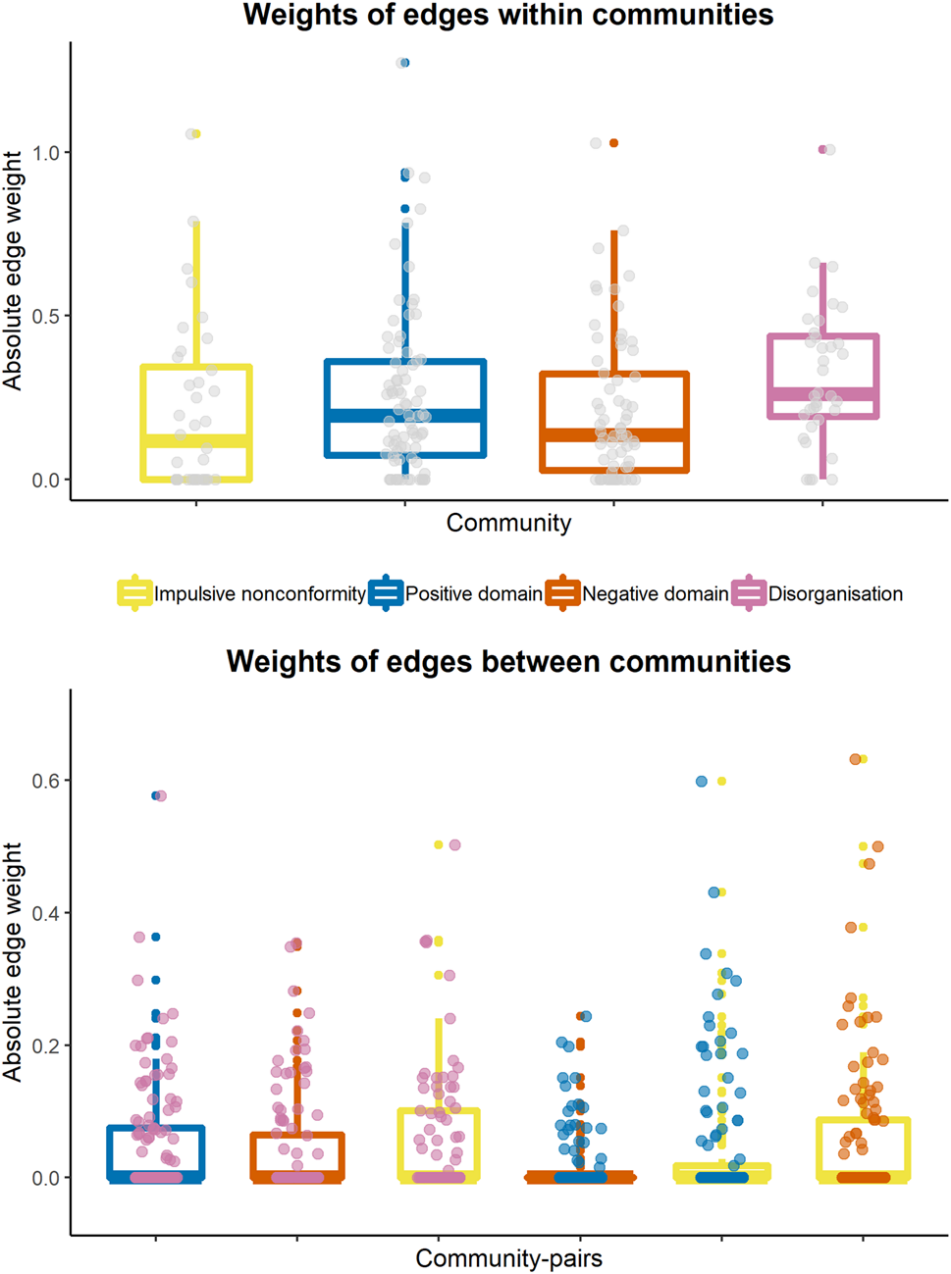
Weights of edges that are located within (top) and between (bottom) communities. In the bottom panel, the colour of the points and the boxplot indicates the community pair whose connection strength is shown. Note that the network is undirected and mapping of community to points vs. boxplots is arbitrary

Finally, we examined differences in absolute edge weights within and between communities (as our aim was to investigate the overall strength of connections within and between communities, we took zero-weight edges into account as well). There was a significant difference in within-community edge weights across communities (Kruskal–Wallis *χ*^2^(3) = 9.87, *p *= 0.02) (Fig. 3, top panel). Follow-up Mann–Whitney tests showed that edge weights within the disorganisation community were significantly larger than edge weights within the impulsive nonconformity (*p* = 0.01, Cliff’s Δ = 0.33) and the negative domain community (*p* = 0.006, Cliff’s Δ = 0.33), and tended to be larger than edge weights within the positive domain community (*p* = 0.056, Cliff’s Δ = 0.23). All the other differences in within-community edge weights were non-significant (all *p* values > 0.13). The edge weights between communities differed significantly across community–community connections (Kruskal–Wallis *χ*^2^(5) = 28.92, *p *< 0.001) (Fig. 3, bottom panel). Follow-up Mann–Whitney tests indicated that weights of edges connecting the positive and the negative community were significantly lower than weights of edges connecting all the other community pairs (all *p* values < 0.021, Cliff’s Δs ranged from 0.11 to 0.29). Additionally, weights of edges connecting the disorganisation and the impulsive nonconformity community were significantly larger than weights of edges between the disorganisation and the positive community (*p* = 0.03, Cliff’s Δ = 0.16). The other between-community edge weights did not differ significantly from each other (all *p* values > 0.067 and all Cliff’s Δs < 0.13).

## Discussion

The aim of this study was to apply novel network modelling techniques to resolve discrepancies with regards to the structure of schizotypy in the sO-LIFE [26, 27], a widely used schizotypy questionnaire. We estimated the network structure of the sO-LIFE in a large general population sample. The network had excellent stability, as shown by split-half and bootstrapping analyses. With a data-driven algorithm, we found network communities that almost perfectly matched the subscales of the sO-LIFE, thus providing substantial support for the psychometric validity of the sO-LIFE. The results validate the classic three-factor model of schizotypy, in that UE, IA and CD were not only separated from each other but could also be differentiated clearly from IN. Thus, our findings imply that it is essentially a theoretical choice whether or not to include IN, but IN does not enter into and dilute the classic three-factor model of schizotypy (e.g. [16, 17, 27]). However, it should be noted that the IN subscale had only modest internal consistency reliability in the present sample, whereas the weight of edges within the IN community was significantly lower only in contrast to the disorganisation community.

Nodes in the disorganisation community had significantly higher closeness centrality, relative to all the other communities. This implies that at the between-person level, when both direct and indirect associations are taken into account, features related to cognitive disorganisation are strongly related to other schizotypal features assessed by the sO-LIFE. Thus, elevated disorganised features in an individual may predict increased positive, negative, and impulsive schizotypy, and vice versa. This result is in line with previous studies on the O-LIFE, reporting that the highest correlations could be found between the CD subscale and the other scales [25, 28]. This pattern has also been shown for other schizotypy questionnaires like the Schizotypal Personality Questionnaire (SPQ) [15]. For example, Gross et al. [20] reported a higher correlation between the disorganised factor and both the cognitive–perceptual (comparable to the UE scale in the O-LIFE) and the interpersonal factor (comparable to the IA scale of the O-LIFE), as compared to the association between the latter two, while in a high-powered study, Christensen et al. [57] found that positive and negative schizotypy showed practically zero correlation (*N* = 6265, *r* = 0.03/*N* = 1000, *r* = 0.01) after controlling for disorganised schizotypy (performing a partial correlation between UE and IA controlling for CD in the present sample returned a highly similar coefficient: *r *= 0.02). Moreover, our results are in line with a recent study showing that in patients with schizophrenia spectrum disorders, cognitive symptoms were the most central in the network including positive, negative and cognitive symptoms and various cognitive functions [74]; our findings also concur with a developmental study that revealed that disorganisation mediated the longitudinal association between negative and positive schizotypy in non-psychotic help-seeking adolescents [75].

Importantly, the disorganisation community reflects cognitive disorganisation, that is, associative loosening, poor attention, language abnormalities and difficulties with decision making [55], which differs somewhat from behavioural disorganisation (odd behaviour and speech) that is measured by the SPQ [15]. Seminal theories of schizophrenia have posited that associative loosening and cognitive slippage are primary, core features of the schizophrenic phenotype [1, 76], while longitudinal studies have shown that impaired attention precedes the emergence of social deficits and positive symptoms [77–82]. There is evidence indicating that cognitive disorganisation (CD) scale scores are related to objectively assessed linguistic and attentional impairment: higher CD has been related to poor performance on tasks assessing vocabulary, similarities, humour and proverbs [83], to reduced sensitivity and prolonged reaction times on the continuous performance task (CPT) [84], and impaired backward visual masking [85]. Moreover, a recent meta-analysis reported that context integration impairment—as assessed by the AX-CPT—correlates positively with disorganised symptoms across the psychosis spectrum [86]. Taken together, we suggest that schizotypy and psychosis high-risk research should pay greater attention to (cognitive) disorganisation, as elevated closeness centrality of CD in our network model implies that high CD is likely to co-occur with the combination of high levels of both positive and negative schizotypy, which is associated with the worst outcomes (e.g. [13, 87, 88]). Little is known, however, about whether CD specifically predicts cognitive deficit or functional impairment over and above (or perhaps even instead of) the effects of positive and negative schizotypy.

We observed no significant differences between communities in terms of betweenness: nodes across communities did not differ in terms of their importance in shortest paths between other nodes (i.e. to what extent they mediate the association of other nodes). Moreover, no significant differences were observed between communities with respect to strength: nodes across the communities did not differ in how strongly they were related to their neighbours (i.e. specific associations with other nodes).

Additionally, weights of edges within the disorganisation community were significantly larger, relative to edge weights within the impulsive nonconformity and the negative schizotypy communities, while the difference relative to edge weights between within the positive schizotypy community was marginally significant. Thus, one may conclude that disorganised features, as assessed by the sO-LIFE, are particularly strongly associated with each other. This might be due to a higher content-related proximity of the disorganised items, especially compared to the items of the negative domain. With regards to the latter, it should be noted that it is of course possible to divide the items into two different subdimensions, one measuring social anhedonia (e.g. Do you like mixing with people?) and one measuring physical anhedonia (e.g. Do you find the bright lights of a city exciting to look at?).

With respect to edges connecting communities, their absolute weights were the smallest between the positive and the negative communities, implying that these are the least related aspects of schizotypy. Interestingly, previous studies on the relationships of the O-LIFE subscales have reported a very similar pattern. For example, Mason and Claridge [28] and Mason et al. [25] found no significant correlations between UE and IA (*r* = 0.09 and *r* = − 0.08). The same pattern is observed for other schizotypy measures: For example, Venables and Rector [89] found no significant association between a positive symptoms scale and scales assessing social or physical anhedonia. In addition, weights of edges between the disorganisation and the impulsive nonconformity community were larger than weights of edges connecting the disorganisation and the positive domain community, suggesting that impulsive nonconformity might partially mediate the association between disorganisation and positive schizotypy.

The network analysis identified three items that did not overlap with the a priori-defined subscales of the sO-LIFE. Especially for two of these, the assignment by the network analysis may not be all that surprising: First, the item Are you usually in an average kind of mood, not too high and not too low? that originally belongs to the IN scale, was assigned to the negative domain. This is somewhat understandable, as an average kind of mood can be easily mistaken for affective flattening, known to be associated with the negative schizotypy dimension [90]. Second, the CD item Do you dread going into a room by yourself where other people have already gathered and are talking? was also assigned to the negative domain. This items may be interpreted as reflecting aspects of social anxiety, which is also known to be a component of negative schizotypy [6]. For future construction of schizotypy questionnaires, it should be considered to no longer include such ambiguous items that are not as appropriate as the other items to differentiate between the different dimensions of schizotypy.

Recently, several studies have examined the network structure of schizotypy or psychotic experiences in large, cross-sectional datasets [53, 54, 59, 91]. However, these were limited either in that they did not use algorithms to detect communities, or the scope of the analysed data was more or less restricted (i.e. affective or cognitive features were absent). In our study, we attempted to overcome these limitations using a data-driven algorithm to segment the network into communities, and we compared the strength of connections within and between these communities to infer how impulsive nonconformity and cognitive disorganisation structurally relate to positive and negative schizotypy. Importantly, our research goes beyond a recent network study of schizotypy in several aspects [54]. First, we analysed data collected with a questionnaire that examines schizotypy from a personality framework (sO-LIFE) as opposed to a clinical approach (SPQ). Second, our network model involved items assessing cognitive disorganisation, a construct of key theoretical importance (discussed above) that is not assessed by the SPQ. Finally, we had a sample recruited from the general population in a single country (Germany), while the study by Fonseca-Pedrero et al. [54] had data from twelve countries, and a large amount of them was obtained in student samples. Thus, our sample is likely to be more heterogeneous in terms of demographic characteristics, and in our study, any confounding by potential differences in item meaning across translations can be ruled out.

The reliability of the results of the present study is strengthened by the large sample size. A limitation of the study is, however, that no further demographic or psychometric data are available on the sample; additionally, no exclusion criteria were applied. In some studies of schizotypal personality, the presence of a psychiatric disorder is an exclusion criterion to rule out actual symptoms causing elevated schizotypy scores. Here, as we did not apply this criterion, individuals who may have received diagnoses of clinical disorders might have participated in the study, thereby possibly inflating between-subject variance. However, schizotypal personality traits have been argued to indicate less severe manifestations of the extended psychosis phenotype [92, 93]. This continuum assumption [5, 6] provides a theoretical rationale for analysing data from participants with and without mental disorders together. Exclusive reliance on self-report (see [94]), absence of information about response rate and the lack of an infrequency scale might have biased the data and can be seen as additional limitations of the study. Furthermore, we note readers that the utility of closeness centrality in psychological networks has recently been questioned [95]. Whilst addressing the majority of the theoretical issues raised by Bringmann et al. [95] is beyond the scope of the present paper, we wish to emphasise that stability analyses suggested that closeness centrality was highly stable in our network model (see Supplementary Material), and also that we do not imply that cognitive disorganisation is clinically more relevant than other dimensions of schizotypy. An additional criterion is that the cross-sectional nature of the data prevents drawing conclusions on causality [42]. Although our network model appeared highly stable, future studies should replicate the findings in different cultures and clinical samples. Moreover, the application of network modelling to longitudinal and experimental datasets would facilitate the understanding of the dynamics and development of schizotypal personality traits.

## Electronic supplementary material

Below is the link to the electronic supplementary material.

Supplementary material 1 (DOCX 436 kb)
