# Supplementary material for: The network structure of schizotypy in the general population
Source: Eur Arch Psychiatry Clin Neurosci. 2019 Oct 23;271(4):635–45. doi: 10.1007/s00406-019-01078-x (PMC8119252; doi:10.1007/s00406-019-01078-x)
Supplement: Supplementary file 1 — Supplementary material 1 (DOCX 436 kb) [file 406_2019_1078_MOESM1_ESM.docx]

**The Network Structure of Schizotypy in the General Population – Supplementary Material**

# European Archives of Psychiatry and Clinical Neuroscience

Bertalan Polner (1), Eliana Faiola (2), Maria F. Urquijo (3), Inga Meyhöfer (2), Maria Steffens (2), Levente Rónai (4), Nikolaos Koutsouleris (3), Ulrich Ettinger (2)

(1) Department of Cognitive Science, Budapest University of Technology and Economics, Budapest, Hungary

(2) Department of Psychology, University of Bonn, Bonn, Germany

(3) Department of Psychiatry and Psychotherapy, University of Munich, Munich, Germany

(4) Institute of Psychology, University of Szeged, Szeged, Hungary

Corresponding author: Bertalan Polner, Budapest 1111, Egry József utca 1., T épület, V. emelet 506, telephone + 36 1 463 1273, fax: +36 1 463-1072, e-mail: [bpolner@cogsci.bme.hu](mailto:bpolner@cogsci.bme.hu)

# Determining network sparsity (controlled by the hyperparameter γ) with split-half stability analysis

The sparsity of the estimated network can be controlled with the a) decision rule of determining edges and b) the hyperparameter γ (Borkulo et al. 2014). As our sample was relatively large, we wanted to keep specificity high and applied the stricter AND-rule for determining edges. The hyperparameter γ controls the degree of penalty on solutions including more edges. We sought to optimize γ by examining the split-half reliability of edge weight, and node strength, closeness and betweenness for 11 potential values of γ in the [0; 1] closed interval. For each γ value, we fitted Ising-models on 1000 pairs of random split-half samples and calculated Spearman ρ rank correlations between edge weight, and node strength, closeness and betweenness of the two networks. Then, we calculated a composite stability score as the sum of Z-transformed correlation coefficients. For each γ, we computed the mean and the 95% confidence interval of this composite stability score. The network in the whole sample was estimated with the highest γ where the mean composite stability score was in the 95% confidence interval of the highest mean composite stability.

The results of split-half stability analyses are presented in Supplementary Figure 1. In general, split-half stability was high, but better stability was achieved with higher values of γ (resulting in sparser networks). The mean composite stability was the highest for networks estimated with γ = 0.8, and the mean composite stability for networks estimated with γ = 1 was in the confidence interval. Therefore, we set γ = 1 when we estimated the network in the whole sample.


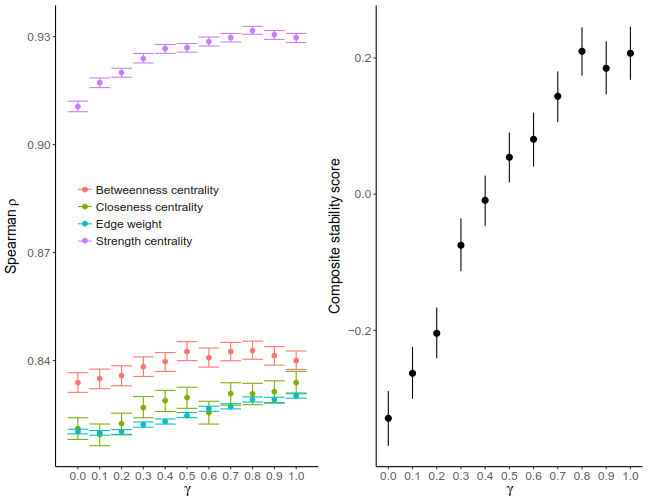


**Supplementary** **Figure 1.** Within-network stability as a function of the γ tuning parameter. We gradually increased gamma from 0 to 1 in steps of 0.1. For each value, the networks were fitted on 1000 random pairs of split-half samples, and Spearman ρ correlation coefficients were calculated as an indicator of within-network stability. In the left panel, the mean±95%CI of the stability estimates for node strength, closeness, and betweenness and edge weights are shown. The right panel shows the mean±95%CI of the composite stability estimates. The stability estimates for node strength, closeness, and betweenness and edge weights were Z-transformed and summed to create a composite network stability score. Networks estimated with γ = 0.8 had the highest mean composite stability, and the 95%CI of this mean included the mean composite stability of networks estimated with γ = 1.

# Network Stability Analyses

The stability of the network estimated in the whole sample was further analysed with bootstrapping methods implemented in the bootnet package (v1.0.1). Using 1000 bootstrap samples, we examined edge-weight accuracy with the nonparametric bootstrap, and centrality stability with the case-dropping bootstrap. We also performed centrality difference tests using the nonparametric bootstrap.

The results of the edge weight accuracy analysis are presented in Supplementary Figure 2. The results of bootstrapped difference tests for node centralities (strength, closeness, betweenness) are shown in Supplementary Figures 3, 4, and 5. The overall pattern of results indicated that there were many significant differences between centralities.


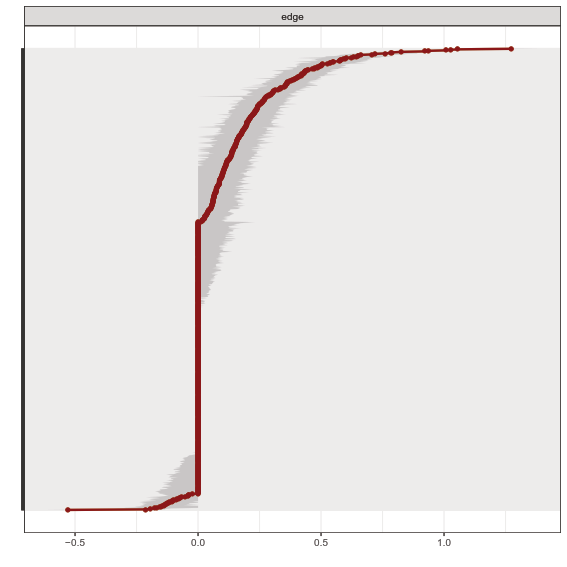


**Supplementary Figure 2.** Bootstrapped confidence intervals (indicated by grey areas) for the estimated edge-weights.


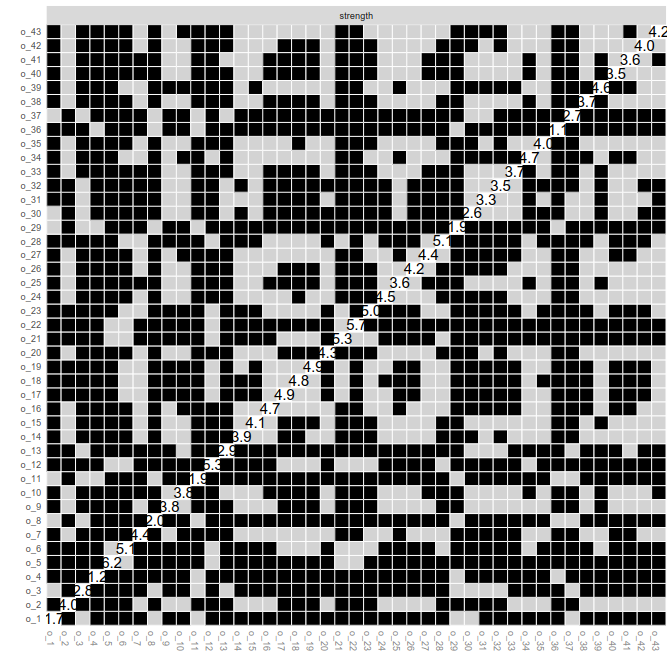


**Supplementary Figure 3.** Bootstrapped difference tests between node strength in the network (α = 0.05). Significant differences are indicated by black boxes. Node strength values are shown at the diagonal. See Table 2. in the main text for the list of O-LIFE items.


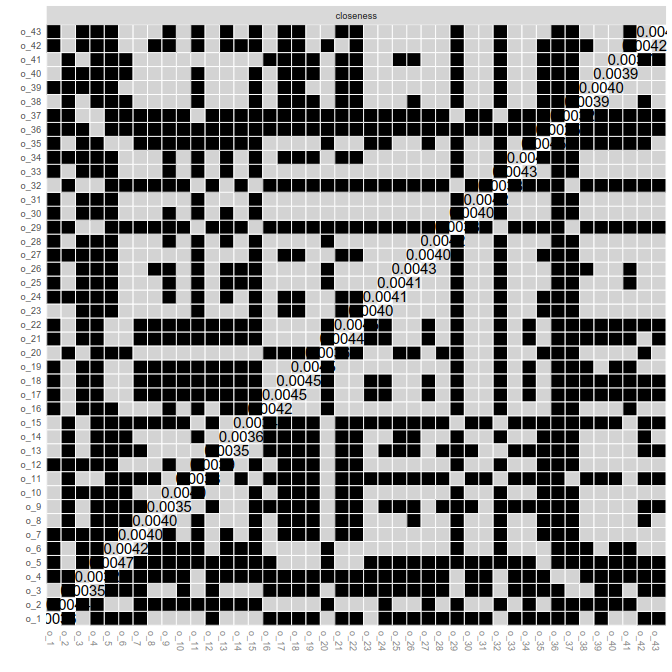


**Supplementary Figure 4.** Bootstrapped difference tests between node closeness in the network (α = 0.05). Significant differences are indicated by black boxes. Node closeness values are shown at the diagonal. See Table 2. in the main text for the list of O-LIFE items.


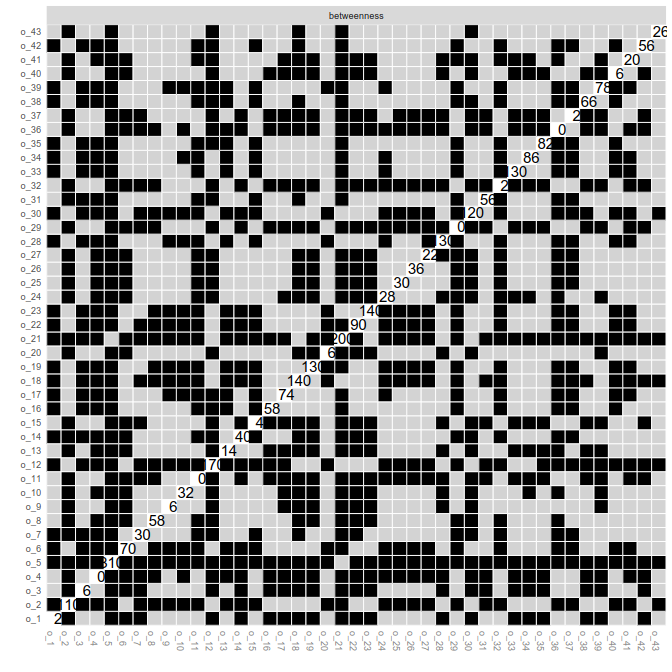


**Supplementary Figure 5.** Bootstrapped difference tests between node betweenness in the network (α = 0.05). Significant differences are indicated by black boxes. Node betweenness values are shown at the diagonal. See Table 2. in the main text for the list of O-LIFE items.

Stability of centralities is presented in Supplementary Figure 6. The analysis indicated that node strength, betweenness and closeness were highly stable in the estimated network: after dropping 60% of cases, 95% of the correlation coefficients between original and bootstrapped node strength, betweenness and closeness were above 0.75.


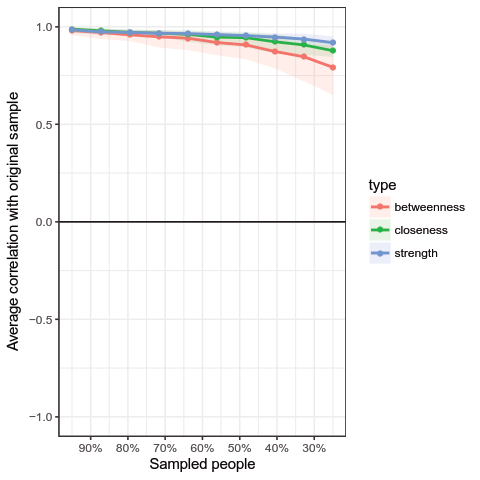


**Supplementary Figure 6.** Estimation of stability of node centralities with case-dropping bootstrap. Lines show the means and areas show the range between the 2.5th and the 97.5th quantiles.

| **#** | **short item** | **full item** | **Sub** | **C** | **CC** | **nCC** | **CCm** |
| --- | --- | --- | --- | --- | --- | --- | --- |
| 1 | Alcohol food | Do you often overindulge in alcohol or food? | IN | Imp | 0.7 | 0 | 0.7 |
| 2 | Difficulty starting | Do you frequently have difficulty in starting to do things? | CD | Dis | 0.72 | 0.22 | 0.64 |
| 3 | Dancing dull | Has dancing or the idea of it always seemed dull to you? | IA | Neg | 0.82 | 0.01 | 0.82 |
| 4 | New foods | Is trying new foods something you have always enjoyed? | IA | Neg | 0.71 | 0 | 0.71 |
| 5 | Enjoy few | Are there very few things that you have ever enjoyed doing? | IA | Neg | 0.78 | 0.24 | 0.71 |
| 6 | Urge harmful shocking | Do you at times have an urge to do something harmful or shocking? | IN | Imp | 0.76 | 0.28 | 0.66 |
| 7 | Almost hears thoughts | Are your thoughts sometimes so strong that you can almost hear them? | UE | Pos | 0.71 | 0.32 | 0.57 |
| 8 | Average mood | Are you usually in an average kind of mood, not too high and not too low? | IN | Neg | 0.62 | 0.19 | 0.53 |
| 9 | Mindreading | Do you think that you could learn to read other's minds if you wanted to? | UE | Pos | 0.79 | 0.28 | 0.71 |
| 10 | Difficulty conversation | When in a crowded room, do you often have difficulty in following a conversation? | CD | Dis | 0.71 | 0.37 | 0.54 |
| 11 | Thinking before doing | Do you stop to think things over before doing anything? | IN | Imp | 0.71 | 0 | 0.71 |
| 12 | Magical powers | Have you ever thought that you had special, almost magical powers? | UE | Pos | 0.8 | 0.35 | 0.7 |
| 13 | Too independent | Are you much too independent to get involved with other people? | IA | Neg | 0.73 | 0.04 | 0.72 |
| 14 | Ideas fast | Do ideas and insights sometimes come to you so fast that you cannot express them all? | UE | Pos | 0.73 | 0.18 | 0.67 |
| 15 | Aware by thinking | Can some people make you aware of them just by thinking about you? | UE | Pos | 0.74 | 0.3 | 0.63 |
| 16 | Thought so real | Does a passing thought ever seem so real it frightens you? | UE | Pos | 0.72 | 0.43 | 0.5 |
| 17 | Mood up and down | Are you a person whose mood goes up and down easily? | CD | Dis | 0.72 | 0.42 | 0.52 |
| 18 | Difficulty keep interested | Do you find it difficult to keep interested in the same thing for a long time? | CD | Dis | 0.74 | 0.34 | 0.61 |
| 19 | Dread going into a room | Do you dread going into a room by yourself where other people have already gathered and are talking? | CD | Neg | 0.72 | 0.29 | 0.6 |
| 20 | Accidents mysterious | Do you feel that your accidents are caused by mysterious forces? | UE | Pos | 0.87 | 0.06 | 0.86 |
| 21 | Mixing with people | Do you like mixing with people? | IA | Neg | 0.86 | 0.16 | 0.83 |
| 22 | Difficulty controlling thoughts | Do you often have difficulties in controlling your thoughts? | CD | Dis | 0.76 | 0.42 | 0.58 |
| 23 | Friends touch | Have you often felt uncomfortable when your friends touch you? | IA | Neg | 0.79 | 0.18 | 0.75 |
| 24 | Urge break smash | Do you ever have the urge to break or smash things? | IN | Imp | 0.72 | 0.34 | 0.57 |
| 25 | Urge injure yourself | Have you ever felt the urge to injure yourself? | IN | Imp | 0.74 | 0.24 | 0.66 |
| 26 | Distracted daydreams | Are you easily distracted from work by daydreams? | CD | Dis | 0.71 | 0.37 | 0.54 |
| 27 | Distracted too much happens | Are you easily confused if too much happens at the same time? | CD | Dis | 0.73 | 0.39 | 0.55 |
| 28 | Vague danger | Do you ever have a sense of vague danger or sudden dread for reasons that you do not understand? | UE | Pos | 0.73 | 0.45 | 0.52 |
| 29 | Massage | Do you love having your back massaged? | IA | Neg | 0.82 | 0 | 0.82 |
| 30 | Average person | Do you consider yourself to be pretty much an average sort of person? | IN | Imp | 0.62 | 0.22 | 0.52 |
| 31 | Other afraid of you | Would you like other people to be afraid of you? | IN | Imp | 0.89 | 0 | 0.89 |
| 32 | Mirror face unusual | When you look in the mirror does your face sometimes seem quite different from usual? | UE | Pos | 0.7 | 0.22 | 0.62 |
| 33 | Shapes in the dark | When in the dark do you often see shapes and forms even though there is nothing there? | UE | Pos | 0.73 | 0.22 | 0.65 |
| 34 | Evil presence | Have you sometimes sensed an evil presence around you, even though you could not see it? | UE | Pos | 0.76 | 0.32 | 0.65 |
| 35 | Hard to make decisions | Is it hard for you to make decisions? | CD | Dis | 0.7 | 0.37 | 0.53 |
| 36 | City lights | Do you find the bright lights of a city exciting to look at? | IA | Neg | 0.68 | 0 | 0.68 |
| 37 | Strong smell | Does your sense of smell sometimes become unusually strong? | UE | Pos | 0.65 | 0.28 | 0.52 |
| 38 | Words mixed up | Do you ever feel that your speech is difficult to understand because the words are all mixed up and don't make sense? | CD | Pos | 0.7 | 0.31 | 0.56 |
| 39 | Do the opposite | Do you often feel like doing the opposite of what other people suggest even though you know they are right? | IN | Imp | 0.7 | 0.34 | 0.55 |
| 40 | Close to friends | Do you feel very close to your friends? | IA | Neg | 0.74 | 0.17 | 0.68 |
| 41 | Spend money | Do you often feel the impulse to spend money which you know you can't afford? | IN | Imp | 0.66 | 0.29 | 0.52 |
| 42 | Distracted read or talk | Are you easily distracted when you read or talk to someone? | CD | Dis | 0.71 | 0.37 | 0.54 |
| 43 | Watch TV or go out | Do you prefer watching television to going out with people? | IA | Neg | 0.78 | 0.18 | 0.74 |

**Supplementary Table 1.** Order of the items of the short O-LIFE in the present study, the short and the full items, the subscale they belong to on the short O-LIFE (Sub), and the community they were assigned to by the algorithm in the present study (C). UE: Unusual Experiences, CD: Cognitive Disorganisation, IN: Impulsive Nonconformity, IA: Introvertive Anhedonia. Imp: impulsive nonconformity, Pos: positive domain, Neg: negative domain, Dis: disorganisation. Node predictabilities were estimated with the mgm package (Haslbeck, 2019). CC: correct classification rate; nCC: normalized correct classification rate; CCm: accuracy of the marginal model.


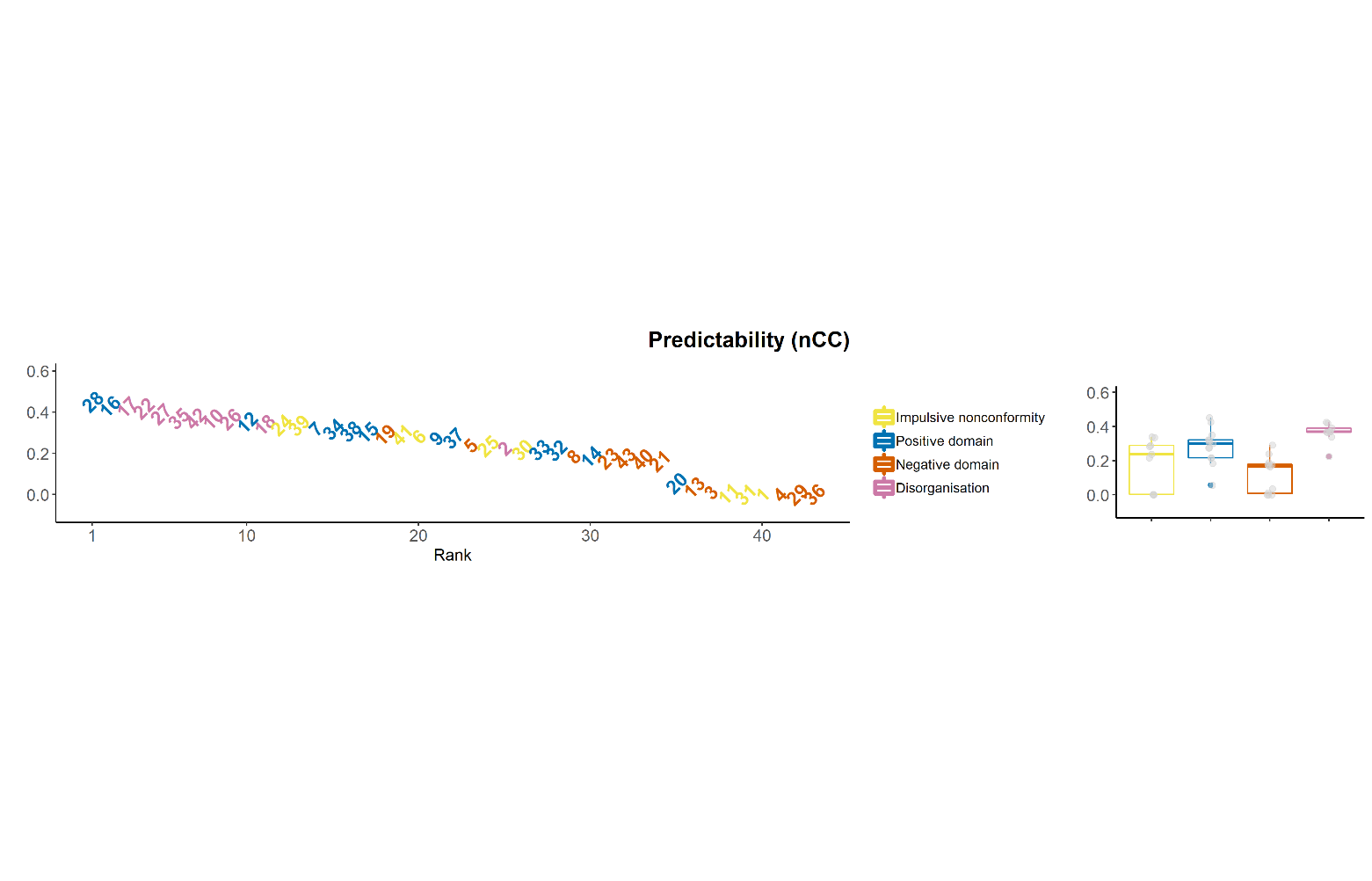


**Supplementary Figure 7.** Distribution of node predictability (normalized correct classification [nCC] rate). On the left side, the nodes are sorted in a descending rank order, while on the right side, the distribution of predictability is shown by community. Predictability significantly differed across communities (Kruskal-Wallis χ^2^(3) = 21.60; *p* < 0.001). Post-hoc tests revealed that nodes in the disorganisation community had higher predictability, relative to nodes in all the other communities (all Mann-Whitney *p* values < 0.033, Cliff’s Δs ranged from 0.56 to 0.96). Moreover, nodes in the positive domain community were more predictable than nodes in the negative domain community (*p* < 0.001, Cliff’s Δ = 0.79). No other post-hoc comparisons were significant (*p* values > 0.17).
